# The Status of First Aid and Its Associations with Health Outcomes among Patients with Traffic Accidents in Urban Areas of Vietnam

**DOI:** 10.3390/ijerph17124600

**Published:** 2020-06-26

**Authors:** Hue Thi Mai, Hai Minh Vu, Tam Thi Ngo, Giang Thu Vu, Huong Lan Thi Nguyen, Men Thi Hoang, Bach Xuan Tran, Carl A. Latkin, Cyrus S. H. Ho, Roger C. M. Ho

**Affiliations:** 1 Institute for Preventive Medicine and Public Health, Hanoi Medical University, Hanoi 100000, Vietnam; huemt93@gmail.com (H.T.M.); ngothitam.tlu@gmail.com (T.T.N.); bach.ipmph@gmail.com (B.X.T.); 2 Department of Trauma, Thai Binh University of Medicine and Pharmacy, Thai Binh 410000, Vietnam; vuminhhai777@gmail.com; 3 Center of Excellence in Evidence-based Medicine, Nguyen Tat Thanh University, Ho Chi Minh City 700000, Vietnam; giang.coentt@gmail.com; 4 Institute for Global Health Innovations, Duy Tan University, Danang 550000, Vietnam; hoangthimen@duytan.edu.vn; 5 Faculty of Nursing, Duy Tan University, Danang 550000, Vietnam; 6 Faculty of Pharmacy, Duy Tan University, Danang 550000, Vietnam; 7 Bloomberg School of Public Health, Johns Hopkins University, Baltimore, MD 21205, USA; carl.latkin@jhu.edu; 8 Department of Psychological Medicine, National University Hospital, Singapore 119074, Singapore; cyrushosh@gmail.com; 9 Department of Psychological Medicine, Yong Loo Lin School of Medicine, National University of Singapore, Singapore 119228, Singapore; pcmrhcm@nus.edu.sg; 10 Institute for Health Innovation and Technology (iHealthtech), National University of Singapore, Singapore 119077, Singapore; 11 Center of Excellence in Behavioral Medicine, Nguyen Tat Thanh University, Ho Chi Minh City 700000, Vietnam

**Keywords:** pre-hospital emergency care, traffic accidents, first aid, Vietnam

## Abstract

While it is well-evident that proper first aid would significantly promote survival and later treatment outcomes, little attention has been paid to improving its capacity in Vietnam. Thus, we conducted this study to assess the status of first aid and its associations with health outcomes among patients in traffic accidents in urban areas of Vietnam. We conducted a cross-sectional study on 413 patients in traffic accidents from October to December 2018 at six hospitals in Thai Binh province. Socio-demographics, first aid characteristics, and health outcomes were collected via face-to-face interviews using a structured questionnaire. We used a chi-square test to determine the differences in health outcomes among those who received first aid and those without. In addition, a multivariable regression was performed to determine the factors associated with first aid. The results indicated that less than half of the patients received first aid (48.1%), and only one fourth received first aid within 10 min after an accident. The proportions of having problems with mobility, self-care, usual activities, and pain/discomfort were significantly lower among those who received first aid compared to those without it. The regression model showed that those with multiple injuries were less likely to receive first aid.

## 1. Introduction

Road traffic accidents are considered to be the leading cause of death globally. According to the World Health Organization (WHO) in 2018, the traffic fatality rate has reached 2.3 million annually [1]. Of note, most cases of road-related deaths have been reported in low- and middle-income countries. Most traffic accidents are time-sensitive illnesses which require proper first aid: immediate assistance before emergency services arrive. Proper first aid is always a prerequisite factor for maximizing survival, mitigating further injuries, and promoting later treatment outcomes. Therefore, strengthening first aid should be the frontline in any efforts aimed to reduce road accident-related morbidities and mortalities. However, in low and middle-income countries with poorly performing healthcare systems and constraint resources, the capacity of pre-hospital care has remained weak. As a result, many injured victims are transported to hospital without live-saving support. 

Vietnam is a country with a diversified transportation system, including motorized two- and three-wheelers, bicycles, cars, and individually constructed vehicles, in which motorcycles are the most commonly used mode of transport, with over 37 million users in 2016. As a result, road accidents are always listed as a leading cause of death with almost one person per hour. WHO estimated that approximately 14,000 people die each year due to road traffic accidents in Vietnam, and this trend has increased over the years [2]. To solve this problem, other than the efforts to reduce the number of road accidents, an effective pre-hospital care system is required, in which first aid should be the frontline. 

In this time of urgent need for the strengthening of pre-hospital care, the Ministry of Health of Vietnam established the national regulation on pre-hospital emergency care in 2008 [3]. Accordingly, each province must establish a pre-hospital emergency center; for provinces that do not have sufficient resources, the pre-hospital emergency team must be incorporated into the provincial hospital. The regulation also emphasized the establishment of a pre-hospital emergency team at district and commune levels. However, very few provinces have such units [4], especially in rural areas [5]. While pre-hospital care services are available in the urban, delay in ambulance dispatch commonly occurs due to mass traffic congestions and a lack of ambulances. As a result, traffic accident victims are commonly transported to hospital by private vehicles without any first aid, boosting the risks of premature deaths and poor treatment outcomes. 

### Problem Statement and Study Objectives

While it is well-evident that proper first aid would significantly promote the chances of survival and later treatment outcomes of patients, little attention has been paid to improving its capacity in Vietnam. Therefore, providing scientific data is essential to help design pragmatic solutions in the future. 

Thus, the study objectives of this study were: (1) to assess the status of first aid and (2) to identify its association with the health among patients in traffic accidents in urban areas of Vietnam. The findings of this study will shape a better understanding of the current status of first aid in Vietnam, enabling policymakers to design more pragmatic solutions to promote first aid in Vietnam. 

## 2. Materials and Methods

**Figure 1:**
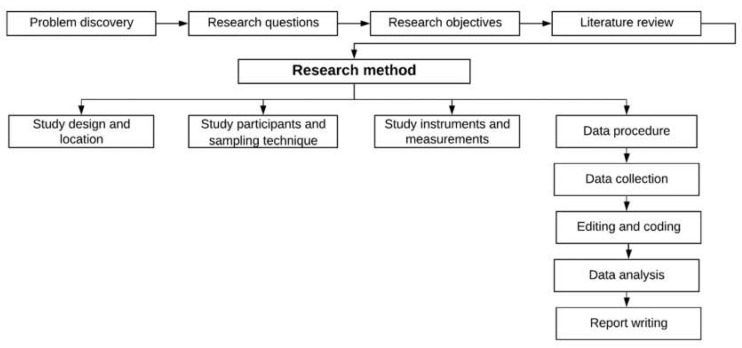
Flowchart of the methodological approach.

The flowchart of methodological approach taken in this study is presented as followed (Figure 1):

### 2.1. Study Design and Location

We conducted a cross-sectional study from October to December 2018 at six hospitals in Thai Binh province, Vietnam, including Kien Xuong, Hung Ha, Dong Hung, Quynh Phu, and Thai Thuy district hospitals, in addition to Thai Binh Provincial General Hospital. Thai Binh is a coastal province in Northern Vietnam. The estimated population in 2019 was almost 1.9 million people, in which 10.6% lived in urban areas and 89.4% lived in rural areas. We purposely selected hospitals located in urban areas.

### 2.2. Study Participants and Sampling Technique

Patients were invited to enroll in the study if they met the following criteria: (1) being at least 18 years old; (2) having traffic accidents; (3) receiving treatment at the chosen hospitals; (4) being able to answer the questionnaire. Those with emergency conditions and severe injuries were excluded from the study. We used a convenience sampling technique to recruit participants. All eligible participants were clearly explained the study purposes and signed a written informed consent form before an interview. There was a total of 413 patients enrolled in this study, in which the majority were males (61.3%), had obtained high school education (81.4%), were a farmer/worker (55.0%), and were urban residents (85.5%). The average age was 45.5 years old (SD = 17.0). 

### 2.3. Study Instruments and Measurements

Socio-demographic characteristics: We collected information on the age, gender, education, marital status, employment, monthly income, and health insurance status of the patients. These findings have been previously reported [6,7].

First aid characteristics: Patients were asked whether they received first aid after the accident. Information regarding the first aid provider, time, and location of first aid was collected. Data on pre-hospital emergency care were also obtained, including respiratory and circulatory supports, spinal fixation, soft-tissue injury, and fracture first aid.

Health outcomes: Information regarding the number of days hospitalized and the hospital admission status of the patients was collected. In addition, psychological distress was measured using the Kessler scale (K6) which developed with support from the U.S. government’s National Center for Health Statistics. This is a tool used for screening mental health issues in a general adult population, including six items. The score of each question ranged from 0 to 4 and the total score ranged from 0 to 24. A Kessler score of >5 was considered as the cut off point for psychological distress. We used the EuroQol-5 Dimensions—5 Levels (EQ-5D-5L) instrument to measure the health-related quality of life (HRQOL) of the participants. This tool comprises of EQ-5D descriptive system and the EQ visual analogue scale (EQ-VAS)-developed by the EuroQol Group in 1987. EQ-5D descriptive system comprises of five dimensions: mobility, self-care, usual activities, pain/discomfort, and anxiety/depression. Each dimension has five options, ranging from 1 (“extreme problem”) to 5 (”no problem”), for the purposes of evaluation. Moreover, self-rated health was evaluated by using EQ-VAS. The scores ranging from 0 (“the worse health condition that you can imagine”) to 100 (“the best health condition that you can imagine”).

### 2.4. Data Procesure

#### 2.4.1. Data Collection

Initial data were collected by well-trained doctors and nurses at selected hospitals using a hard-copy questionnaire. All eligible patients were invited to a private room in the hospital for the interview, which lasted 10–15 min. Information regarding the socio-demographics, injury-related characteristics, first aid, HRQOL, and level of psychological distress was collected. For clinical characteristics that could not be obtained from the patients, we extracted such data from their medical records, after asking for permission from the patients.

Before the commencement of fieldwork, a pilot study was undertaken in 50 patients to refine the study questionnaire. It is generally suggested that a preliminary survey requires a minimum sample size of 30 participants for a pilot study [8].

#### 2.4.2. Editing and Coding

As the project was implemented, the database was built using Epidata (Version 3.1, Statens Serum Institut, Copenhagen, Denmark) and regularly cleaned by experienced data managers. Data were stored in a password-secured computer and only used for the study purposes.

#### 2.4.3. Data Analysis

We used STATA^®^ version 15.0 (Stata Corp. LP, College Station, Brazos County, TX, USA) to analyze the data. Descriptive analysis was used to summarize the social-demographic and first aid characteristics. We used a chi-square test to determine the differences in health outcomes among those who received first aid and those without it; these included the quality of life, psychological distress, and number of days hospitalized of the patients. Both a univariate regression (Equation (1)) and a multivariable regression (Equation (2)) were performed to determine the factors associated with first aid. The equations for these statistical analyses were as followed:

(1) $$Y_{t}=\beta_{1}+\beta_{2}X_{t}+u_{t}$$

in which $Y_{t}$ is the dependent variable that varies as $X_{t}$ does, $\beta_{1}$ is the intercept, $\beta_{2}$ is the slope of the intercept line, $X_{t}$ is the explanatory variable, and $u_{t}$ is the random component of the regression handling the residue; and

(2) $$log\left[\frac{P}{1-P}\right]=\beta_{0}+\beta_{n}\left(A_{n}\right)$$

in which (*P*/1 − *P*) is the odd ratio, $\beta_{0}$ is the intercept, $\beta_{n}$ are the slopes, and $A_{n}$ refers to explanatory variables. Independent variables included the role in the accident, accident site, area of the accident, light conditions, transport purpose, types, and the number of wounds/ injuries; the outcome variable was whether or not patients received first aid. We selected variables with *p*-values of log-likelihood ratio test <0.2 from univariate regressions for the final model. A *p*-value of less than 0.05 was considered statistically significant. 

### 2.5. Ethical Approval

We obtained approval from the Institutional Review Board of the Thai Binh University of Medicine and Pharmacy to carry out this study (Decision no. 7642/HĐĐĐ).

## 3. Results

Table 1 indicates that less than half of the patients received first aid (48.1%). Among these, 56.7% received first aid from medical staff. Notably, only 26.1% received first aid within 10 min of the accident (66.5%). In addition, only one-third of patients received first aid at the scene of the accident (31.5%). Prehospital emergency care was rarely performed, including respiratory support (1.5%), cervical spine fixation (4.0%), and circulatory support (18.2%). First aid for soft-tissue wounds and fractures were more commonly performed.

Table 2 illustrates the differences in health outcomes between those who received first aid and those without it. The proportion of patients who had problems with mobility (*p* = 0.01), self-care (*p* = 0.01), usual activities (*p* < 0.05), and pain/discomfort (*p* < 0.05) were significantly lower among those who received first aid, compared to those without it. There were no significant differences in psychological distress, number of days hospitalized, Kessler-6 score, and EQ VAS score between those received first aid and those without it. 

The results of the regression model are summarized in Table 3. Those with soft tissue injuries (OR = 1.74, 95% CI: 1.12; 2.69), maxillofacial injuries (OR = 2.91; 95% CI: 1.15; 7.34), and fractures (OR = 2.51, 95% CI: 1.53; 4.11) were more likely to receive first aid. In contrast, those with multiple injuries (OR = 0.46, 95% CI: 0.26; 0.82) were less likely to receive first aid.

## 4. Discussion

Our study aimed to describe the status of first aid and assess its associations with health outcomes among patients in traffic accidents in urban areas of Vietnam. The results showed that less than half of the respondents received first aid, and only 26.1% received first aid within 10 min of the accident. First aid, such as respiratory support, cervical spine fixation, and circulatory support, was rarely performed. In regard to its associations with health outcomes, the proportions of patients having problems with mobility, self-care, usual activities, and pain/discomfort were significantly lower among those who received first aid compared to those without it. The regression model showed that those with multiple injuries were less likely to receive first aid.

We observed the low percentage of patients receiving first aid. In low-income countries, the majority of patients were carried to the hospital by their relatives or went there by themselves. Vietnam is among countries that have an ineffectiveness of pre-hospital emergency care. Although Vietnam has a national emergency number (115) and national regulations on pre-hospital emergency care were established in 2008, which emphasized the establishment of a 115 emergency center in provincial hospitals, only big cities have this center [3]. As a result, the majority of road accident patients are transported by a private car or taxi, without receiving proper first aid. Since a large number of early deaths are attributable to inadequate pre-hospital care [9,10], we highlight the urgent need for more effective pre-hospital care to meet the growing demand of the population.

The result showed that only 26.1% received first aid within 10 min of the accident. This percentage was much lower than data reported from Thailand. An initial review of data from Khon Kaen, Thailand, suggested that 90% of cases received first aid within 10 min [11]. This may be attributable to a lack of proper preparedness and response plan in Vietnam. Furthermore, the lack of first aid might increase the risk of post-traumatic stress disorders and impaired HRQOL [7]. Given the fact that time is a crucial element to prevent early death and improve patient outcomes [12], it is important to invest more efforts into promoting preparedness and response plans for pre-hospital emergency care. This can be done by regular training for doctors and nurses and increasing the number of ambulances. More importantly, we should develop strong networks of well-trained first responders so that they can promptly provide first aid for victims while waiting for the EMS (Emergency Medical System) team. This approach has been successfully implemented in other low-resource settings such as Thai Lan [13].

Notably, life-saving procedures, including respiratory/circulatory support and cervical spine fixation, were rarely performed. These are complicated procedures and require specialized equipment. However, given the poor accessibility of pre-hospital emergency care services in Vietnam, many victims are directly transported to the hospital without respiratory/circulatory support and/or cervical spine fixation. As a result, many patients may be vulnerable to preventable death and poor treatment outcomes. We call for more effective investments in pre-hospital emergency care services, including the areas of human resources, equipment, and operation, to meet the increasing demand of the population. 

It is notable that problems with mobility, self-care, usual activities, and pain/discomfort were more commonly reported in patients who did not receive first aid. There has been a growing body focused on the impacts of pre-hospital care on treatment outcomes. A study by Konstantin Klein et al. suggested that that survival after severe trauma is largely attributable to the quick performance of life-saving measures in pre-hospital settings [14]. A prospective observational study by Johannes found a positive association between pre-hospital care and rates of survival to hospital admission [15]. However, evidence on the link between pre-hospital care and quality of life of hospitalized patients has not been fully understood. Although our study could not draw the casual relationships between pre-hospital care and quality of life, we provided premilitary evidence on its relationship which could provide a basis for future research. Under the scope of this study, we reaffirm the importance and significance of proper pre-hospital care in improving the health outcomes of patients.

We observed that those with multiple injuries were less likely to receive first aid. In Vietnam, it is very common that unskilled bystanders provide first aid for accident victims and transport them to the hospital. Notably, most of these bystanders do not have any basic first aid skills. As a result, in extremely severe cases, bystanders may not be willing to provide first aid for victims. Thus, it is crucial to implement community-based education programs to strengthen their first aid skills. Such programs should prioritize schools or community organizations, such as the Youth Union.

The most important implication of our study is the need to strengthen pre-hospital care. This can be done by designing a unified model of pre-hospital care which can be nationally applied in Vietnam. In addition, healthcare providers should receive adequate training to provide quick response in severe situations. At a national level, it is essential to establish a policy structure and make use finances wisely to strengthen pre-hospital care in Vietnam.

Several limitations should be noted of this study. First, this study focused on first aid, rather than pre-hospital care, so we did not obtain data on response time, which is defined as the time between notification of an occurrence and the ambulance arrival at the scene [16]. Given its fundamental component of assessing the performance of prehospital care, we recommend future researchers include this indicator when assessing the capacity of pre-hospital emergency care. In addition, alcohol consumption is well-recognized as a major factor which is precipitant for road traffic accidents in Vietnam [6]. This study did not explore alcohol consumption before road traffic accidents, which could further impair the cognition and verbal expression of patients and might result in a delay to first aid. According to interviewing proxies, we were not able to interview family members, so we were required to exclude unconscious and life-threatening patients from the interview stage. This may induce the risk of selection biases because people with more severe injuries might obtain quicker medical care. Nevertheless, it is almost impossible to recruit such patients in our study, due to the unavailability of a first aid surveillance system Vietnam. Thus, it is important for future researches to quantify the number of patients excluded due to life-threatening conditions to measure the effects of this on the study results. 

## 5. Conclusions

To sum up, our study suggested that a lack of first aid was commonly observed in traffic accident patients in urban areas of Vietnam. Essential life support was rarely performed, and those with multiple injuries were less likely to receive first aid. The results highlight the urgent need for the establishment of emergency centers at provincial levels. In addition, it is also important to develop networks of well-trained first responders and to strengthen their skills with qualified education programs.

## Figures and Tables

**Table 1:** First aid characteristics for people in road accidents.

First Aid Characteristics	*n*	%
**Received first aid**		
No	210	50.9
Yes	203	48.1
**Time from the accident to the first aid**		
<10 min	53	26.1
10–60 min	135	66.5
>60 min	15	7.4
**First aid site**		
The scene	64	31.5
Commune health facilities	58	28.6
District health facilities	73	36.0
Others	8	3.9
**First aid provider**		
Herself/himself	4	2.0
Bystanders	84	41.4
Medical staff	115	56.7
**Respiratory supports**		
Oxygen therapy	3	1.5
None	200	98.5
**Cervical spine fixation**		
Fixed brace	7	3.5
Others	1	0.5
None	195	96.1
**Circulatory support**		
Infusion NaCl 0.9%	36	17.7
Infusion NaCl and High molecular weight solution	1	0.5
None	166	81.8
**First aid for a soft-tissue wound**		
Simply wash	66	32.5
Simple bandages	43	21.2
Tape pressed	25	12.3
Hemostasis	18	8.9
Tourniquet	3	1.5
None	48	23.7
**First aid for fracture**		
Bone fixation brace	69	34.0
Motionless	18	8.9
Fixed with a soft cloth	29	13.3
Motionless on a hard stretcher	3	1.5
None	84	41.4

**Table 2:** Differences in health outcomes between those who received first aid and those without it.

Characteristics	Did Not Receive First Aid		Received First Aid		Total		*p*-Value
	*n*	%	*n*	%	*n*	%	
Having problems with mobility	183	87.1	158	77.8	341	82.6	**0.01**
Having problems with self-care	190	90.5	166	81.8	356	86.2	**0.01**
Having problems with usual activities	182	86.7	159	78.3	341	82.6	**<0.05**
Having problems with pain/discomfort	203	96.7	187	92.1	390	94.4	**<0.05**
Having problems with anxiety	205	97.6	192	94.6	397	96.1	0.11
Psychological distress	28	13.3	29	14.3	57	13.8	0.78
Being unconscious during hospitalization	14	6.7	7	3.5	21	5.1	0.14
	**Mean**	**SD**	**Mean**	**SD**	**Mean**	**SD**	
Number of days hospitalized	7.65	4.5	7.44	3.6	7.53	4.0	0.97
Kessler-6 score	2.64	2.4	2.88	2.3	2.76	2.6	0.63
EQ VAS	66.6	16.2	63.3	18.2	65	17.3	0.10

EQ-VAS: the EQ visual analogue scale, SD: standard deviation.

**Table 3:** Factors associated with receiving first aid.

Characteristics	Received First Aid			
	Univariate Regression		Multivariable Regression	
	OR ^a^	95% CI	OR ^b^	95% CI
**Role in the accident** (vs. accident causers)				
Accident victims	1.23	0.62; 2.41		
Self-accident	0.88	0.43; 1.76		
**Accident site** (vs. highway)				
Urban road	1.21	0.58; 2.50		
Rural road	1.02	0.55; 1.88		
Others	1.09	0.19; 5.93		
**Area of accident** (vs. city)				
Town	1.70	0.84; 3.40	1.93	0.89; 4.14
Delta area	0.89	0.47; 1.64	0.97	0.48; 1.96
**Light conditions** (vs. bright)				
Dark, sufficient street-lights	0.96	0.49; 1.88	0.67	0.31; 1.40
Dark, insufficient street-lights	1.34	0.78; 2.28	1.32	0.73; 2.36
Dark, no street-lights	1.64	0.78; 3.45	1.78	0.76; 4.11
**Transport purpose** (vs. passenger transport)				
Cargo delivery	0.55	0.16; 1.85		
None	1.18	0.54; 2.53		
Others	2.00	0.49; 8.08		
**Types of wounds/injuries**				
Soft-tissue injuries yes vs. no)	1.74 *	1.12; 2.69	1.89 *	1.13; 3.15
Hand injuries (yes vs. no)	0.82	0.31; 2.12		
Traumatic brain injuries (yes vs. no)	1.15	0.69; 1.88		
Maxillofacial injuries (yes vs. no)	1.91	0.82; 4.44	2.91 *	1.15; 7.34
Spine injuries (yes vs. no)	0.50	0.18; 1.37	0.71	0.23; 2.15
Chest injuries (yes vs. no)	0.20 *	0.04; 0.93	0.23	0.04; 1.22
Fractures (yes vs. no)	2.41 **	1.57; 3.68	2.51 **	1.53; 4.11
Multiple injuries (yes vs. no)	0.34 **	0.19; 0.56	0.46 **	0.26; 0.82
**Number of wounds/injuries** (vs. None)				
1	0.54	0.18; 1.54		
≥2	1.10	0.36; 3.36		

CI: Confident Interval; ^a^ Crude Odd ratio; ^b^ Adjusted Odd ratio; ** *p* < 0.01, * *p* < 0.05.
